# The impact of migration characteristics on rural migrant households’ farmland use arrangements in China

**DOI:** 10.1371/journal.pone.0273624

**Published:** 2022-08-29

**Authors:** Chong Lu, Ailin Wu

**Affiliations:** 1 Research Institute of Social Development, Southwestern University of Finance and Economics, Chengdu, Sichuan, China; 2 School of Public Finance and Taxation, Southwestern University of Finance and Economics, Chengdu, Sichuan, China; Shenzhen University, CHINA

## Abstract

This paper investigates the impacts of migration characteristics on rural migrant households’ farmland use arrangements in China. The results reveal that trailing migration, duration of migration and the proportion of co-migrants have a significant effect on the probability of rural migrant households’ farmland abandonment. Commercial employment migration has a negative impact on the abandonment of farmland by migrant families. Migrant households are most likely to choose farmland abandonment in the western and middle regions of China and in small farmland areas. In the eastern region, and first tier and second tier Chinese cities, migrant households are more inclined to choose farmland transfer. Household earnings increase, which induces households to gradually give up the cultivation of farmland or to transfer farmland, constituting a mechanism in Chinese households’ farmland use arrangements. Notably, the consolidation of arable land should be the focus in areas of low economic development. Furthermore, an effective mechanism for the transfer of farmland should be established.

## 1 Introduction

Farmland use pattern change is a phased phenomenon brought about by the transfer of rural labor to cities in developing countries, such as Latvia, Ecuador, St. Lucia, Nepal, Poland, Pakistan, China, etc. [1–5]. In China, significant changes in rural land use patterns brought about by mass migration are taking place [6] and the most prominent changes concern farmland abandonment and transfer. China’s rural laborer migration has been increasing for a long time.

Migration and land use patterns have a strong correlation [7], and the different stages of labor migration lead to different land use structures [8]. Cropland abandonment was driven by migration and not caused by land fragmentation in Albania [9]. Migrants cannot combine off-farm work with farm work, so migration reduces the levels of chemical inputs used in agricultural production [10, 11], which leads to reduced income from agricultural production. This will also lead to a decline in soil fertility [12]. Based on the threshold model, labor migration will significantly promote rural land transfer if it is less than or equal to 0.125, but when it is greater than 0.125, its impact on land transfer is not significant [13].

The factors affecting land use patterns are many and varied. Farmland use intensity decreased with the increase of rural out-migration in Guangdong Province, China from 1996 to 2012. And the higher the ratio of migrant labor income to household income, the greater the probability farmers will choose land transfer or abandonment [14]. The age of 60 as the new rural pension scheme eligibility threshold is associated with large increases in land rented out in China [15]. Applying the DD method and the PSM-DD method, the rural land titling program had a positive effect on households’ land-related investment [16]. Additionally, administrative restrictions on the free flow of labor and land hinder agriculture restructuring, forcing rural migrant families to abandon their farmland in China [17]. land certification increases the land renting-out by less productive farmers and the land renting-in by more productive farmers [18]. Under the influence of local planning and policy implementation, in China, the transformation of regional land-use types leads to an increased strain on limited land resources and frequent conflicts between various land-use types [19].

Some studies have discussed how migration affects land use arrangements in China [7]. By employing data from a survey in the mountainous areas of Sichuan Province, the higher the ratio of migrant labor income to total household income, the greater the probability that farmers choose land abandonment [14]. By employing survey cross-sectional data from inside China, with every 10% increase in off-farm employment and part-time employment, the average probability of rural households’ farmland abandonment increases by 4% and 5%, respectively [20]. By adopting a two-stage least squares estimator and a recursive mixed-process model, seasonal and permanent migration negatively affects land efficiency but with no significant difference between the two effects [21]. Notably, our research finds that off-farm employment has a significant positive effect on farmland abandonment only in the western regions of China. On the contrary, off-farm employment has a significant negative effect on farmland abandonment in the eastern and middle regions of China.

Only a few studies focus on farmland decisions for the different types of rural labor migration in China. Indeed, existing research is limited to one province or region, and some studies only discuss that population migration will promote farmland abandonment. Additionally, the mechanisms at work between farmland use arrangements and rural migration characteristics are less discussed. For China as a whole, there is shortage of up-to-date and authoritative empirical evidence on the relationship between different reasons for migration, the duration of migration and the proportion of co-migrants, and the farmland use arrangements. In this paper, based on the household head’s migration reasons (off-farm employment, business employment and trailing migration), the duration of migration and the proportion of co-migrants, the relationship between household migration characteristics and Chinese rural migrants’ household farmland use arrangements is analyzed. The mechanism will also be further developed.

The efficiency of the allocation of labor resources (rural and urban) is constrained by the barriers to population movement presented by the household registration system in China. Furthermore, labor migration between townships and cities restricts the allocation efficiency of China’s land resources. Analyzing the relationship between household migration characteristics and migrant household farmland use arrangements is meaningful for three reasons. Firstly, it can provide policy advice for the reform of China’s farmland system, and provide a theoretical basis for improving the allocation efficiency of China’s farmland resources and labor resources. Secondly, it can provide empirical evidence for improving Chinese rural household welfare. Thirdly, our study contributes to the literature on migration and farmland use arrangements in developing countries. Traditional theories of rural-urban migration do not consider the role of farmland in the process of rural-urban migration. Most studies place their focus on the characteristics of farmland use arrangements [22, 23].

The contributions of this paper are as follows: (1) Based on the latest and authoritative cross-sectional data on rural migration households in China, the relationship between off-farm employment, business employment, trailing migration, the duration of migration and the proportion of co-migrants, and the farmland use arrangements of migrant households are systematically studied. (2) This paper explores the mediating role of household earnings on the relationship between household migration characteristics and household farmland use arrangements. (3) From the perspective of household migration characteristics, the trends of labor migration and farmland use arrangements in China are studied, and new empirical evidence is provided on these matters.

The remainder of the paper is structured as follows. Section 2 describes the background, data and variable definitions. Section 3 presents the theoretical framework and empirical model. Section 4 presents the basic results of the analysis of migration characteristic effects and the heterogeneity analysis of the results. Section 5 presents the mechanical analysis of the results. Section 6 and section 7 offer conclusions and policy implications.

## 2 Background, data and variable definitions

### 2.1 Farmland use arrangements and migration in China

Table 1 shows the farmland use arrangements and migration in China. For farmland abandonment, the higher proportion of farmland abandonment is mainly in the provinces of western and eastern China, such as Ningxia Province, Chongqing Province, Zhejiang Province, Fujian Province, and Guizhou Province. Of the above sample provinces, we can find that the highest farmland abandonment rates reach 28.81% in Ningxia province. For farmland use for farming, the higher proportion of farmland used for farming is mainly in the provinces of eastern and middle of China, such as Shandong Province, Hebei Province and Henan Province. Similarly, the fourth Column shows the distribution of the proportion of farmland transfer. The higher proportion of farmland transfer is mainly in the provinces of western and eastern China, such as Heilongjiang Province, Jilin Province and Zhejiang Province. In addition, the provinces with higher migrant laborer rates in China are also mainly in the western and central regions. In these provinces with a high proportion of household migrant laborers, we can find that the household migration laborer rate reaches 10.12%, 9.94% and 8.65% in Anhui Province, Henan Province and Sichuan Province, respectively.

**Table 1:** Farmland use arrangements and migration in China.

Region	Farmland abandonment	Farmland use for farming	Farmland transfer	Ratio of rural household migration
China	0.0677	0.5562	0.3761	100.00
Beijing	0.0000	0.6667	0.3333	0.00
Tianjin	0.0000	0.6786	0.3214	0.04
Hebei	0.0212	0.7055	0.2733	4.62
Shanxi	0.0548	0.6613	0.2839	2.21
Inner Mongolia	0.0516	0.5182	0.4302	2.96
Liaoning	0.0079	0.6479	0.3442	1.55
Jilin	0.0038	0.4582	0.5380	2.15
Heilongjiang	0.0048	0.3755	0.6197	3.72
Shanghai	0.0000	0.5000	0.5000	0.00
Jiangsu	0.0131	0.5542	0.4327	3.02
Zhejiang	0.1355	0.4295	0.4350	1.72
Anhui	0.0357	0.5046	0.4597	10.12
Fujian	0.1319	0.5198	0.3483	2.34
Jiangxi	0.0887	0.4515	0.4598	3.96
Shandong	0.0097	0.7149	0.2754	6.89
Henan	0.0126	0.6624	0.3250	9.94
Hubei	0.0674	0.5345	0.3981	4.17
Hunan	0.0816	0.4835	0.4349	6.15
Guangdong	0.1168	0.5444	0.3388	1.07
Guangxi	0.0689	0.6326	0.2985	2.95
Hainan	0.1263	0.6053	0.2684	0.26
Chongqing	0.1993	0.4074	0.3933	4.14
Sichuan	0.1125	0.4936	0.3939	8.65
Guizhou	0.1220	0.5394	0.3386	3.73
Yunnan	0.0808	0.5941	0.3251	3.24
Tibet	0.0161	0.8629	0.1210	0.17
Shaanxi	0.1156	0.6349	0.2495	3.75
Gansu	0.0867	0.6428	0.2706	3.73
Qinghai	0.1113	0.4988	0.3900	1.09
Ningxia	0.2881	0.3955	0.3164	1.15
Xinjiang	0.0027	0.5829	0.4144	0.51

Note: The value for each province is the average of the sum of the cities in the sample.

### 2.2 Data and variables

This paper uses data from the China Migrants Dynamic Survey (CMDS) Project in 2017. The CMDS is a cross-sectional survey conducted since 2009. The survey was conducted by the Migrant Population Service Center, National Health Commission P.R. China. To ensure that the sample was nationally representative, the CMDS covered 31 provinces (municipalities) of mainland China. A multistage cluster, stratified, probability proportional to size (PPS) sampling method was used. The annual sample size is nearly 160,000. The content covers basic information, mobility and trends of migrants and family members, employment and social security, income and expenditure, basic public health services, marriage, and so on. Here, we use the 2017 data, because only this year’s data covers the information on rural migrant household farmland use arrangements. The all-sample distribution is shown in Table 2.

**Table 2:** Spatial distributions of sample provinces.

Region	Ration of residence	Region	Ration of residence
Beijing	3.17	Hubei	2.63
Tianjin	3.21	Hunan	3.69
Hebei	3.81	Guangdong	3.64
Shanxi	2.29	Guangxi	2.74
Inner Mongolia	3.07	Hainan	0.81
Liaoning	2.77	Chongqing	3.11
Jilin	2.12	Sichuan	3.44
Heilongjiang	2.01	Guizhou	3.27
Shanghai	3.15	Yunnan	4.22
Jiangsu	5.61	Tibet	0.95
Zhejiang	7.17	Shaanxi	3.43
Anhui	3.89	Gansu	2.62
Fujian	4.15	Qinghai	2.47
Jiangxi	2.21	Ningxia	1.99
Shandong	5.01	Xinjiang	3.30
Henan	4.06		

Our dataset includes rural migrant households in China, excluding urban migrant households and all the observations of missing values in any independent or dependent variables. After processing, there are 73,373 observations in our sample. Note that the number of observations differs across different model specifications.

The core dependent variables are the farmland use arrangements of rural migrant households. This study discusses three types of farmland use arrangements for rural households: farmland abandonment, farming and transfer. We add a series of control variables, such as the household head’s age, level of education, the distance from the hometown to the destination city, the annual of household income, etc. This will help us reduce estimation bias.

Table 3 presents the summary statistics for the dependent and explanatory variables. Overall, about 6.8% of rural Chinese migrant households choose farmland abandonment; approximately 55.6% choose farmland cultivation; and 37.6% choose farmland transfer. More prominently, 61.3% of China’s rural migrant households migrate for off-farm work, and 26.6% migrate for business employment. The average duration of migration is 6.501 years. And the average age of the head of the migrant household is 37.35.

**Table 3:** Summary statistics.

Variables	Meaning and measurement	Mean	SD
Abandonment	If the household abandons farmland (0 = no;1 = yes)	0.068	0.251
Farming	If the household farms farmland (0 = no;1 = yes)	0.556	0.497
Transfer	If the household transfers farmland (0 = no;1 = yes)	0.376	0.484
Off-farm employment	Migration reason (off-farm employment = 1; others = 0)	0.613	0.487
Business employment	Migration reason (business employment = 1; others = 0)	0.266	0.442
Trailing migration type	Migration reason (trailing household member migrations = 1; others = 0)	0.121	0.326
The duration of migration	The time from the time of migration to 2017 (year)	6.501	6.151
Proportion of co-migrants	Ratio of household members who move with household head. (%)	0.815	0.255
Gender	Gender of household head (0 = femal;1 = male)	0.571	0.495
Age	Age of household head (year)	37.35	10.143
Education	Education level of household head. (Under primary school degree = 1; primary school degree = 2; middle school degree = 3; high school degree = 4; junior college degree = 5; undergraduate degree = 6; postgraduate degree = 7)	3.187	1.011
Medical insurance	Whether the head of the household is covered by medical insurance (0 = no;1 = yes)	1.237	0.456
Number of elderly members	The number of 64 + year-old household members (number)	0.033	0.209
Household size	Total number of household members (number)	3.320	1.171
Household income	The total per-month income of the household (RMB/ person)	2284.756	1811.662
Land size	Rural household’s land area (Mu)	1.894	2.365
Job status	Whether have work. (0 = no;1 = yes)	0.862	0.345
Distance	Distance between migration destination and hometown (1 = Inter-County in one city; 2 = Inter-city in one province; 3 = Inter-province)	1.711	0.773

*Notes*: The results in the table are derived from statistical analysis of the samples; One dollar equal to 6.8918 RMB in 2017. One Mu equal to 0.0667 Hectares.

## 3 Theoretical framework and empirical model

In China, where the economy is developing quickly, the migration of rural labor directly brings about an increase in non-agricultural income [11, 24]. In addition, the reduction of the number of households engaged in agricultural production will directly lead to the decline of agricultural production efficiency, and the pattern of farmland utilization will gradually change [25, 26]. Under the new economics of labor migration, we investigate the relationship between household migration characteristics and farmland use arrangements [27–29].

This study has three dependent variables: farmland abandonment, farmland farming and farmland transfer. Ordinary least squares (OLS) regression is adopted to explore the impact of migration characteristics on migrant household farmland use arrangements. The model we use to estimate is:

$${farmland\_use}_{hc}=\alpha +\beta {migration}_{hc}+\delta X_{hc}+\varphi H_{hhc}+\lambda_{c}+\mu_{hc}.$$ (1)

Where the dependent variable *farmland*_use_*hc*_ represents the outcome of interest, and the subscript *hc* indicates the outcome for household *h* from county *c*. The key explanatory variable *migration*_*hc*_ is the actual value for household *h*.

*X*_*hc*_ is a vector of household characteristics including the household size, land size, number of elderly members, and so on. *H*_*hhc*_ represents a group of household head’s characteristics including age, education, and so on.

*λ*_*c*_ is the city fixed effect. Adding *μ*_*hc*_ into the regression equation helps us remove the effects of city-level characteristics, such as special local labor policies. Furthermore, the city-level effect helps us remove all unobserved or omitted characteristics for each city. For instance, it helps us control for peasant workers’ preference for cities. Furthermore, by including *λ*_*c*_ in the regression equation, we can control for different effects due to culture and social conventions.

*μ*_*hc*_ is the household specific error term. In addition, taking into account the intra-cities correlation of the error term, we cluster the standard errors at the city level for all regressions.

## 4 Empirical results

### 4.1 Basic analysis

Table 4 shows the impact of migration characteristics on migrant household farmland use arrangements, using an OLS regression. The average marginal effects calculated after the estimation are listed in columns (1) ‒ (5). Here we only report the key explanatory variables’ results after controlling for household head, household characteristics and city fixed effects. The regression results for all variables are reported in A1 Appendix of S1 Appendix.

**Table 4:** Baseline results.

Model 1: Farmland abandonment	(1)	(2)	(3)	(4)	(5)
Off-farm employment	0.002				
	(0.002)				
Business employment		-0.007***			
		(0.002)			
Trailing migration			0.009***		
			(0.004)		
Duration of migration				0.003***	
				(0.001)	
Proportion of co-migrants					0.022***
					(0.002)
Model 2: Farmland farming	(6)	(7)	(8)	(9)	(10)
Off-farm employment	0.056***				
	(0.004)				
Business employment		-0.027***			
		(0.005)			
Trailing migration			-0.088***		
			(0.007)		
Duration of migration				-0.018***	
				(0.002)	
Proportion of co-migrants					-0.152***
					(0.004)
Model 3: Farmland transfer	(11)	(12)	(13)	(14)	(15)
Off-farm employment	-0.057***				
	(0.004)				
Business employment		0.034***			
		(0.004)			
Trailing migration			0.079***		
			(0.006)		
Duration of migration				0.016***	
				(0.001)	
Proportion of co-migrants					0.130***
					(0.004)
Household characteristics	YES	YES	YES	YES	YES
Cities fixed effects	YES	YES	YES	YES	YES
Observations	73,373	73,373	73,373	73,373	73,373

*Notes*: The values in parentheses are the values of the city level robustness standard errors for each variable. Significance codes

***p<0.01 **p<0.05 *p<0.1.

Model 1 shows the relationship between the indicators of migration characteristics and farmland abandonment. Among them, the relationship between off-farm employment and farmland abandonment is not significant. Business employment has a negative effect on farmland abandonment, and is statistically significant at the 1% level. In contrast, when other conditions remain unchanged, for every 1% increase in trailing migration, the average migrant households’ farmland abandonment increases by 0.9%. Comparing the duration of migration, the proportion of co-migrants in the rural household has a similar positive effect on farmland abandonment, and is statistically significant at the 1% level. Specifically, keeping all other variables constant, for every 1% increase in duration of migration and in the proportion of co-migrants in the household, the probability of farmland abandonment increases by 0.3% and 2.2%, respectively. And these estimates are also statistically significant at the 1% level.

We observe that the coefficient for migration characteristics is significant at the 1% level in model 2. Among them, with every 1% increase in off-farm employment, the probability of farming farmland increases by 5.6%. In contrast to off-farm employment, there is a negative and significant relationship between business employment, trailing migration, duration of migration, the proportion of co-migrants of rural household and farmland farming. Specifically, when all other variables remain unchanged, for every 1% increase in business employment, trailing migration, the duration of migration and the proportion of co-migrants in households, the average probability of farmland farming will decrease by 2.7%, 8.8%, 1.8% and 15.2%, respectively.

Model 3 presents the relationship between migration characteristics and farmland transfer. In column (1), it can be seen that for every 1% increase in off-farm employment, the average probability of farmland transfer will decrease by 5.7%. In columns (2)-(5), we find similar results. When all other conditions remain unchanged, for every 1% increase in business employment, trailing migration, duration of migration and the proportion of co-migrants in the rural household, the average probability of farmland farming will decrease by 3.4%, 7.9%, 1.6% and 13%, respectively. We also find that these estimates are statistically significant at the 1% level.

### 4.2 Robustness analysis

In this section, we perform several robustness checks to increase confidence in our main results. Table 5 presents the average marginal effects results. To sum up, the results are consistent with the baseline results.

**Table 5:** Robustness analysis results.

Variables	Abandonment (1)	Farming (2)	Transfer (3)
Model 1: Probit model			
Off-farm employment	0.001	0.158***	-0.168***
	(0.016)	(0.010)	(0.011)
Business employment	-0.056***	-0.076***	0.102***
	(0.019)	(0.012)	(0.012)
Trailing migration	0.104***	-0.256***	0.226***
	(0.026)	(0.017)	(0.017)
Time of migration	0.032***	-0.057***	0.049***
	(0.006)	(0.004)	(0.004)
Proportion of co-migrants	0.180***	-0.423***	0.376***
	(0.020)	(0.013)	(0.013)
Model 2: Logit model			
Off-farm employment	0.019	0.254***	-0.272***
	(0.033)	(0.017)	(0.017)
Business employment	-0.128***	-0.123***	0.167***
	(0.038)	(0.019)	(0.019)
Trailing migration	0.192***	-0.418***	0.367***
	(0.053)	(0.028)	(0.029)
Time of migration	0.069***	-0.092***	0.079***
	(0.013)	(0.007)	(0.007)
Proportion of co-migrants	0.374***	-0.691***	0.620***
	(0.041)	(0.021)	(0.022)
Model 3: Different cluster			
Off-farm employment	0.002	0.056***	-0.057***
	(0.003)	(0.005)	(0.005)
Business employment	-0.007**	-0.027***	0.034***
	(0.003)	(0.006)	(0.006)
Trailing migration	0.009**	-0.088***	0.079***
	(0.004)	(0.008)	(0.007)
Time of migration	0.003***	-0.018***	0.016***
	(0.001)	(0.002)	(0.002)
Proportion of co-migrants	0.022***	-0.152***	0.130***
	(0.003)	(0.005)	(0.005)
Control variables	YES	YES	YES
Counties fixed effects	YES	YES	YES
Observations	73,373	73,373	73,373

*Notes*: In the model 3 robust standard errors are in parenthesis which are clustered at the cities level. The values in parentheses are the values of the robust standard errors for each variable. Significance code

***p<0.01

**p<0.05

*p<0.1.

Firstly, considering the dependent variables are binary variables, then the binary Probit and Logit models were respectively adopted to investigate the impact of migration characteristics on farmland use arrangements. The results given by models 1–2 are highly consistent with the results of the benchmark regression. We can draw the conclusion that the migration characteristics are significantly related to rural migrant household farmland use arrangements. As shown in models 1 and 2, if the head of the household’s migration reason is business employment, the household is more likely to abandon farmland. More specifically, if the head of the household’s migration reason is trailing migration, the household will be more likely to abandon farmland. And if the duration of the household head’s migration is longer, the probability of choosing to abandon farmland is higher. Similarly, if the proportion of co-migrants is higher, the household is more likely to choose land abandonment. Secondly, off-farm employment is significantly positively correlated to household choosing farmland farming. And the business employment, trailing migration, duration of migration, and the proportion of co-migrants are all significantly negatively related to choosing to farm the farmland. Additionally, the relationship between off-farm employment and farmland transfer is significantly positive. The correlations between business employment, trailing migration, duration of migration, and the proportion of co-migrants are all significantly positively related to farmland transfer.

Secondly, considering the intra-group correlation between samples, this paper clustered the standard errors into the county-level and used the most stringent criteria to test the robustness of the results. The results of model 3 show that the coefficient is still significant at the 1% level, indicating that the relationship between migration characteristics and farmland use arrangements is robust.

### 4.3 Heterogeneity analysis

The previous study considered all household migrant laborers as having the same preference for farmland use arrangements. In this section, we first introduce heterogeneity of household heads, focusing on the level of education of the household head, and the role of the age of the household head in farmland abandonment. Then, we introduce the heterogeneity of the household, focusing on the total income of the household, and the role of household farmland area in farmland abandonment. Finally, we introduce the heterogeneity of the city, focusing on the location of the cities and the role of cities’ rank level in farmland abandonment. In addition, in A2 Appendix of S1 Appendix, the regression results for farmland farming and transfer are reported.

#### 4.3.1 Household head heterogeneity

Table 6 illustrates the heterogeneity analysis results. Columns (1) and (2) show the average marginal effect between migration characteristics and farmland abandonment for household heads’ education level. The relationship between migration characteristics and farmland abandonment for household heads with a low education level is significantly correlated. And these regression results are consistent with the baseline results. Compared with household heads with a high education level, a household head engaged in off-farm employment will not choose to abandon farmland. However, a household head engage in business employment will likely choose to abandon farmland. Columns (3) and (4) show the average marginal effect between migration characteristics and farmland abandonment for household heads below and above the age of 35. It is worth noting that for household heads older than 35, the number of trailing migrants, the duration of migration and the proportion of co-migrants increase, and the probability of farmland abandonment increases.

**Table 6:** The heterogeneous analysis results—farmland abandonment.

Variables	Higher education	Lower education	Age (> = 35)	Age (<35)	Larger farmland
	(1)	(2)	(3)	(4)	(5)
Off-farm employment	-0.015***	0.006**	-0.003	0.005*	0.004
	(0.004)	(0.003)	(0.003)	(0.003)	(0.004)
Business employment	0.011**	-0.009***	-0.002	-0.005*	-0.006
	(0.004)	(0.003)	(0.004)	(0.003)	(0.004)
Trailing migration	0.017***	0.003	0.009*	-0.002	0.000
	(0.006)	(0.005)	(0.005)	(0.006)	(0.006)
Time of migration	0.008***	0.003*	0.008***	0.002	0.002
	(0.002)	(0.001)	(0.002)	(0.001)	(0.002)
Proportion of co-migrants	0.017***	0.021***	0.024***	0.017***	0.018***
	(0.004)	(0.003)	(0.004)	(0.003)	(0.004)
Variables	Smaller farmland	Higher income	Lower income	Eastern region	Middle region
	(6)	(7)	(8)	(9)	(10)
Off-farm employment	0.000	-0.005	0.004	-0.007*	-0.009**
	(0.003)	(0.003)	(0.003)	(0.004)	(0.004)
Business employment	-0.007**	0.002	-0.010***	0.003	0.004
	(0.003)	(0.004)	(0.003)	(0.004)	(0.005)
Trailing migration	0.014***	0.012*	0.008*	0.014*	0.014**
	(0.005)	(0.006)	(0.004)	(0.007)	(0.007)
Time of migration	0.004***	0.002	0.005***	0.003	0.005**
	(0.001)	(0.002)	(0.001)	(0.002)	(0.002)
Proportion of co-migrants	0.019***	0.010**	0.024***	0.012***	0.011**
	(0.003)	(0.005)	(0.003)	(0.004)	(0.005)
Variables	Western region	1^st^ tier cities	2^nd^ tier cities	3^rd^ tier cities	4^th^ tier cities
	(11)	(12)	(13)	(14)	(15)
Off-farm employment	0.013***	0.002	-0.001	-0.013**	0.010***
	(0.005)	(0.007)	(0.003)	(0.006)	(0.004)
Business employment	-0.018***	-0.001	-0.005*	0.008	-0.014***
	(0.005)	(0.008)	(0.003)	(0.007)	(0.004)
Trailing migration	0.004	-0.004	0.015***	0.020*	0.003
	(0.008)	(0.011)	(0.006)	(0.010)	(0.006)
Time of migration	0.007***	0.000	0.003*	0.002	0.006***
	(0.003)	(0.003)	(0.002)	(0.003)	(0.002)
Proportion of co-migrants	0.032***	0.003	0.013***	0.021***	0.030***
	(0.005)	(0.007)	(0.003)	(0.007)	(0.004)
Control variables	YES	YES	YES	YES	YES
Cities fixed effects	YES	YES	YES	YES	YES

*Notes*: Robust standard errors are in parenthesis which are clustered at the cities level. All regressions control for the household head, household variables and cities fixed effects. The values in parentheses are the values of the robust standard errors for each variable. Significance code

***p<0.01

**p<0.05

*p<0.1.

#### 4.3.2 Household heterogeneity

Columns (5)-(8) demonstrate the average marginal effect between migration characteristics and farmland abandonment for household in terms of household heterogeneity. Overall, it can be seen that when the household farmland area is small, and the income level of household is low, the relationship between migration characteristics and household farmland abandonment is consistent with the baseline results [30].

#### 4.3.3 City heterogeneity

Columns (9)-(15) report the average marginal effect between migration characteristics and farmland abandonment for households in terms of city heterogeneity [31]. Overall, the duration of migration and the proportion of co-migrants are significantly positively related to rural household farmland abandonment in western and middle regions of China. And off-farm employment is significantly negatively related to farmland abandonment in eastern and middle regions of China. However, off-farm employment is significantly positively related to farmland abandonment, in western regions of China [9]. Additionally, the duration of migration, the proportion of co-migrants, and trailing migration are significantly positively related to farmland abandonment in the 2^nd^-4^th^ tier cities of China.

## 5 Mechanism analysis

**Fig 1:**
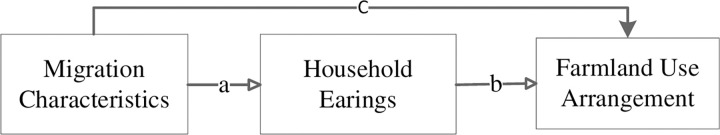
The mediating relationship.

Table 4 confirms the impact of migration characteristics on farmland use arrangements. Household earnings are considered to be a key factor in household migration decisions and farmland use arrangements [32–35]. The mechanism is shown in Fig 1. Then, household earnings have a mediating effect on the household migration characteristics and the farmland use arrangements. This paper examines the existence of this mediating effect by introducing an interaction term between the household migration characteristics and household earnings in the benchmark regression model.

We estimate the following equation:

$$\begin{array}{l} {farmland\_use}_{hc}=\alpha +\beta {earning}_{hc}+\gamma {migration}_{hc}\times {earning}_{hc}+\delta X_{hc}\\ \;+\varphi H_{hhc}+\lambda_{c}+\mu_{hc}. \end{array}$$ (2)

Where the dependent variable *farmland*_use_*hc*_ represents household farmland use arrangements: farmland abandonment, farming and transfer, and the subscript *hc* indicates the outcome for households *h* from cities *c*. The key explanatory variable *earning*_*hc*_ is the actual value for households *h*. *migration*_*hc*_×*earning*_*hc*_ is the interaction term. The meanings of variables *X*_*hc*_, *H*_*hhc*_, *λ*_*c*_, *μ*_*hc*_ are the same as in Eq (1).

Table 7 demonstrates the effects of household migration characteristics on farmland use arrangements in terms of household earnings, that is, the mediating effect of household earnings, using an OLS regression. The average marginal effects calculated after the estimation are listed in columns (1) ‒ (5) of model 1- model 3. Column (2) of model 1 shows that the business employment coefficient is significantly negative and the interaction term coefficient is significantly positive. This means that the impact of business employment on farmland abandonment decreases with the increase of household earnings. Similarly, columns (1), and (3)-(5) of model 1 report off-farm employment, trailing migration, the duration of migration and the proportion of co-migrants are significantly positive and the interaction term coefficients are significantly negative. This means that the impact of off-farm employment, trailing migration, the duration of migration and the proportion of co-migrants on farmland abandonment decreases with the increase of household earnings.

**Table 7:** Mediating effect analysis results.

Variables		Model1: Farmland abandonment			
	(1)	(2)	(3)	(4)	(5)
Income	-0.013***	-0.020***	-0.017***	-0.009**	-0.024***
	(0.004)	(0.004)	(0.004)	(0.004)	(0.005)
Off-farm employment	0.054**				
	(0.025)				
Off-farm employment * Income	-0.007**				
	(0.003)				
Business employment		-0.104***			
		(0.026)			
Business employment * Income		0.013***			
		(0.003)			
Trailing migration			0.088**		
			(0.044)		
Trailing migration * Income			-0.010*		
			(0.006)		
Time of migration				0.042***	
				(0.009)	
Time of migration* Income				-0.005***	
				(0.001)	
Proportion of co-migrants					0.146***
					(0.028)
Proportion of co-migrants* Income					-0.017***
					(0.004)
Intercept	0.135***	0.186***	0.203***	0.109***	0.217***
	(0.029)	(0.028)	(0.027)	(0.029)	(0.034)
Variables		Model 2: Farmland Farming			
	(6)	(7)	(8)	(9)	(10)
Income	-0.025***	0.010**	-0.028***	-0.018***	-0.008**
	(0.004)	(0.004)	(0.006)	(0.006)	(0.003)
Off-farm employment	-0.078**				
	(0.038)				
Off-farm employment * Income	0.014***				
	(0.005)				
Business employment		0.141***			
		(0.046)			
Business employment * Income		-0.019***			
		(0.006)			
Trailing migration			-0.160**		
			(0.063)		
Trailing migration * Income			0.015*		
			(0.008)		
Time of migration				-0.031**	
				(0.015)	
Time of migration* Income				0.004*	
				(0.002)	
Proportion of co-migrants					-0.167***
					(0.055)
Proportion of co-migrants* Income					0.014**
					(0.007)
Intercept	0.983***	0.633*	1.155***	1.132***	0.746**
	(0.031)	(0.338)	(0.042)	(0.048)	(0.334)
Variables		Model 3: Farmland transfer			
	(11)	(12)	(13)	(14)	(15)
Income	0.040***	0.043***	0.055***	0.054***	0.019***
	(0.007)	(0.006)	(0.006)	(0.007)	(0.007)
Off-farm employment	-0.097**				
	(0.045)				
Off-farm employment * Income	0.005				
	(0.006)				
Business employment		0.026			
		(0.049)			
Business employment * Income		0.001			
		(0.006)			
Trailing migration			0.072		
			(0.069)		
Trailing migration * Income			0.003		
			(0.009)		
Time of migration				0.069***	
				(0.017)	
Time of migration* Income				-0.007***	
				(0.002)	
Proportion of co-migrants					0.410***
					(0.050)
Proportion of co-migrants* Income					-0.038***
					(0.007)
Intercept	-0.325***	-0.376***	-0.474***	-0.461***	-0.222***
	(0.050)	(0.048)	(0.046)	(0.052)	(0.051)
Observations	73,169	73,169	73,169	73,169	73,169

*Notes*: Robust standard errors are in parenthesis which are clustered at the cities level. The values in parentheses are the values of the robust standard errors for each variable. Significance code

***p<0.01

**p<0.05

*p<0.1.

As shown in columns (6) and (8)-(10) of model 2, off-farm employment, trailing migration, the duration of migration and the proportion of co-migrants are significantly negative and the interaction term coefficients are significantly positive. This means that the impact of off-farm employment, trailing migration, the duration of migration and the proportion of co-migrants on farmland farming decreases with the increase of household earnings. Column (7) of model 2 reports that business employment is significantly positive and the interaction term coefficient is significantly negative. This means that the impact of business employment on farmland farming decreases with the increase of household earnings.

In addition, column (14) of model 3 displays the duration of migration coefficient is significantly positive and the interaction term coefficient is significantly negative. This implies when the level of household earnings increases, the positive effect of the duration of migration on farmland transfer decreases. Notably, column (15) of model 3 also shows the proportion of co-migrants is significantly positive and the interaction term coefficient is significantly negative. This also implies when the level of household earnings increases, the positive effect of the proportion of co-migrants on farmland transfer decreases.

We next measure the size of the mediating effect of household earnings. We use Baron & Kenny’s framework to represented by three structural equations as follows [36]:

$${farmland\_use}_{hc}=\alpha_{1}+\beta_{1}{migration}_{hc}+\delta_{1}X_{hc}+\varphi_{1}H_{hhc}+\lambda_{1c}+\mu_{1hc}.$$ (3)

$${migration}_{hc}=\alpha_{2}+\beta_{2}{earning}_{hc}+\delta_{2}X_{hc}+\varphi_{2}H_{hhc}+\lambda_{2c}+\mu_{2hc}.$$ (4)

$$\begin{array}{l} {farmland\_use}_{hc}=\alpha_{3}+\beta_{3}{earning}_{hc}+\gamma {migration}_{hc}+\delta_{3}X_{hc}+\varphi_{3}H_{hhc}\\ \;+\lambda_{3c}+\mu_{3hc}. \end{array}$$ (5)

Where the dependent variable *farmland*_use_*hc*_ represents household farmland use arrangements, and the subscript *hc* indicates the outcome for households *h* from counties *c*. The key explanatory variable *earning*_*hc*_ is the actual value for household *h*. The meanings of variables *X*_*hc*_, *H*_*hhc*_, *λ*_1*c*_, *μ*_1*hc*_, *λ*_2*c*_, *μ*_2*hc*_, *λ*_3*c*_, *μ*_3*hc*_ are the same as in Eq (1).

Table 8 shows the mediating effect of household earnings and the ratio of mediating effects to the total effect. Overall, the mediation effect accounts for between 0.3442% and 5.5276% of the total effect. Notably, from the absolute value of the mediating effect, the mediating effect of household earnings has the greatest impact on farmland abandonment when the proportion of co-migrants increases. Similarly, the mediating effect of household earnings is the largest on farmland farming when the proportion of co-migrants increases. Additionally, the mediating effect of household earnings is the largest on farmland transfer when the proportion of co-migrants increases. From the relative value of the mediating effects, when the type of migration is trailing migration, the mediating effect has a greater impact on farmland abandonment, farmland cultivation and farmland transfer.

**Table 8:** Mediating effect analysis results–Sobel-Goodman test.

Types		Farmland abandonment			
	Off-farm employment (1)	Business employment (2)	Trailing migration type (3)	Time of migration (4)	Proportion of co-migrants (5)
Indirect effect	-0.0001**	-0.0001**	0.0007***	0.0000	-0.0011***
	(0.0000)	(0.0000)	(0.0001)	(0.0000)	(0.0002)
Direct effect	0.0029	-0.0107***	0.0161***	0.0016**	0.0210***
	(0.0019)	(0.0021)	(0.0033)	(0.0007)	(0.0023)
Total effect	0.0028	-0.0108***	0.0168***	0.0016**	0.0199***
	(0.0019)	(0.0021)	(0.0033)	(0.007)	(0.0023)
Indirect effect/Total effect (%)	[3.5714]	[0.9259]	[4.1667]	[0.0000]	[5.5276]
		Farmland farming			
	Off-farm employment (6)	Business employment (7)	Trailing migration type (8)	Time of migration (9)	Proportion of co-migrants (10)
Indirect effect	-0.0002**	-0.0002**	0.0013***	0.0001	-0.0011***
	(0.0000)	(0.0000)	(0.0003)	(0.0001)	(0.0003)
Direct effect	0.0583***	-0.0235***	-0.1098***	-0.0182***	-0.1498***
	(0.0038)	(0.0043)	(0.0064)	(0.0015)	(0.0045)
Total effect	0.0581***	-0.0237**	-0.1085***	0.0182***	-0.1509***
	(0.0032)	(0.0042)	(0.0064)	(0.0015)	(0.0044)
Indirect effect/Total effect (%)	[0.3442]	[0.8439]	[1.1982]	[0.5495]	[0.7290]
		Farmland transfer			
	Off-farm employment (11)	Business employment (12)	Trailing migration type (13)	Time of migration (14)	Proportion of co-migrants (15)
Indirect effect	0.0004**	0.0004**	-0.0019***	-0.0001	0.0021***
	(0.0001)	(0.0001)	(0.0003)	(0.0000)	(0.0003)
Direct effect	-0.0613***	0.0342***	0.0937***	0.0167***	0.1288***
	(0.0037)	(0.0042)	(0.0063)	(0.0014)	(0.0044)
Total effect	-0.0609***	0.0346***	0.0918***	0.0167***	0.1309***
	(0.0037)	(0.0042)	(0.0063)	(0.0014)	(0.0044)
Indirect effect/Total effect (%)	[0.6568]	[1.1561]	[2.0697]	[0.5988]	[1.6043]
Observations	73,169	73,169	73,169	73,169	73,169

*Notes*: The values in parentheses are the values of the standard errors for each variable. The values in brackets are fraction of indirect effect in total effect. Significance code

***p<0.01

**p<0.05

*p<0.1.

All household head, household variables and cities fixed effect are control in columns (1)-(15).

## 6 Discussion

This study systematically reviewed China’s rural household farmland use arrangements considering different household migration characteristics. Previous studies have shown that non-farm employment or part-time employment will promote farmland abandonment [9, 14]. But we only found this relationship exists in the western region of China. The effects of labor migration on land transfer only exist in some regions or within a certain scale of labor migration [18]. More importantly, existing studies do not tell us about the relationship between the duration of migration, the proportion of co-migrants and farmland use arrangements [32]. This study proves that with the gradual increase of the duration of migration and the proportion of co-migrants, the probability of farmland abandonment increases by 0.3% and 2.2%, respectively. This is one of the important marginal contributions of this study.

It is noteworthy that this study finds that the duration of migration and the proportion of co-migrants has a negative effect on the farming of farmland, however, the household farmland transfer probability will increase, which issue has not been addressed in existing studies [20]. We think the main reason behind this is that the agricultural labor force of rural households is reduced, so the probability of farmland being cultivated is reduced. Furthermore, agricultural cooperative organizations in China have begun to transfer the farmland of the out-migrating population (Fransen et al., 2017). In general, we see that when the duration of migration and the proportion of co-migrants increases by 1%, the probability of farmland transfer increases by 1.6% and 13%, respectively.

Regions, cities and households are all significantly different in China. To address this, this study analyzed the relationship between household migration characteristics and farmland use arrangements from the perspectives of household farmland area, the regions of cities and the tier level of the cities and so on. Previous studies show that small areas of farmland are more likely to be abandoned [20, 25, 37]. This study further discovered that migrants leaving the western and middle regions of China and small farmland areas are most likely to choose farmland abandonment. Additionally, migrants leaving the eastern region and 1^st^ tier and 2^nd^ tier cities are more inclined to choose farmland transfer. These findings give us clear objectives for the governance of China’s cultivated land.

Worth mentioning is that the studies by Che, Nguyen et al., Qian, Wang, & Zheng and Gao et al. only investigated the relationship between laborer migration and farmland use arrangements [12, 17, 18, 32]. The mechanism behind the relationship was not investigated. Although, when income from off-farm work is more than that earned from farming, farmland abandonment is inevitable [38]. Unfortunately, they did not provide empirical evidence. In this study, we have proved the fact using the relevant data. Overall, household income and its interaction coefficient with household migration characteristics is significantly positive for farmland farming. However, household income and its interaction coefficient with household migration characteristics is significantly negative for farmland abandonment. Therefore, when the characteristics of household migration change, the negative effect of household income will be further weakened for households’ farming behavior. At the same time, this study calculated that the mediating effect of household income was about 0.3442% - 5.5276%.

Overall, the contributions of this study are as follows: Firstly, we explore household farmland use arrangements (farmland abandonment, farming and transfer) from the perspective of household migration characteristics [13, 15, 20, 21]. Secondly, we explore the relationship between the duration of migration, the proportion of co-migrants and the household farmland use arrangements, which been neglected in the literature. The study on China in this paper enriches the literature on changes of farmland use arrangements in the process of urbanization in developing countries.

## 7 Conclusions and policy implications

### 7.1 Conclusions

This study investigated the effects of household migration characteristics on migration household farmland use arrangements in China. Overall, more off-farm employment type migration leads to a higher probability of choosing farmland farming. On the contrary, more business employment-type migration leads to a lower propensity for farmland abandonment. Meanwhile, compared with farmland farming, for the business employment and trailing type of migration, the longer the duration and proportion of co-migrants, the more inclined households are to choose farmland transfer. Additionally, the more trailing migration, duration of migration and the proportion of co-migrants leads to a higher propensity for farmland abandonment.

The heterogeneity of abandoned farmland by Chinese migrating households is prominent. Among them, migrants leaving the western and middle regions and small farmland areas are most likely to opt for farmland abandonment. Additionally, those leaving the eastern region, and 1^st^ tier and 2^nd^ tier Chinese cities are more inclined to choose to transfer farmland. After rural laborers migrate, household earnings are increased, which makes households gradually give up the cultivation of farmland or choose to transfer farmland, which may be an important mechanism behind Chinese migrating households’ farmland use arrangements.

### 7.2 Policy implications

This study provides some policy reference for reform of China’s households’ farmland. Firstly, the consolidation of arable land should be the focus in areas of low economic development, in small farmland areas, and in remote areas of China. Furthermore, the scale of arable land use should be expanded to increase the income derived from it. An effective mechanism for the transfer of farmland should be established, so that rural households after migration can transfer the farmland, obtain economic benefits and prevent abandonment. Additionally, China should establish a paid transfer system of farmland, and provide funds to aid migration of rural laborers to cities. This way, the economic value of farmland assets would be realized. Furthermore, this paid transfer system of farmland will better utilize agricultural resources (especially land).

It should be noted that this study has several limitations. The data used in the analyses are short-term data, although they contain rich information on household migration characteristics. In addition, the mechanism behind the impact of migration on farmland use arrangements may be influenced by other factors, such as skills transformation, human capital accumulation [39] and so on. So, future research can further explore these areas.

## Supporting information

S1 Appendix (DOCX)
