# Supplementary material for: The impact of migration characteristics on rural migrant households’ farmland use arrangements in China
Source: PLoS One. 2022 Aug 29;17(8):e0273624. doi: 10.1371/journal.pone.0273624 (PMC9423677; doi:10.1371/journal.pone.0273624)
Supplement: S1 Appendix — (DOCX) [file pone.0273624.s001.docx]

## **Appendix** A1 The basic results of including all variables

| Variables |  | Farmland abandonment | |  |  |
| --- | --- | --- | --- | --- | --- |
|  | (1) | (2) | (3) | (4) | (5) |
| Off-farm employment | 0.002 |  |  |  |  |
|  | (0.002) |  |  |  |  |
| Business employment |  | -0.007*** |  |  |  |
|  |  | (0.002) |  |  |  |
| Trailing migration |  |  | 0.009** |  |  |
|  |  |  | (0.004) |  |  |
| Time of migration |  |  |  | 0.003*** |  |
|  |  |  |  | (0.001) |  |
| Proportion of co-migrants |  |  |  |  | 0.022*** |
|  |  |  |  |  | (0.002) |
| Head gender | -0.000 | -0.000 | 0.001 | -0.000 | 0.000 |
|  | (0.002) | (0.002) | (0.002) | (0.002) | (0.002) |
| Head age | -0.000 | 0.000 | 0.000 | -0.000 | 0.000 |
|  | (0.000) | (0.000) | (0.000) | (0.000) | (0.000) |
| Head education | -0.010*** | -0.010*** | -0.010*** | -0.010*** | -0.009*** |
|  | (0.001) | (0.001) | (0.001) | (0.001) | (0.001) |
| Medical insurance | 0.012*** | 0.011*** | 0.012*** | 0.011*** | 0.012*** |
|  | (0.002) | (0.002) | (0.002) | (0.002) | (0.002) |
| Older | 0.013** | 0.013** | 0.013** | 0.014*** | 0.015*** |
|  | (0.005) | (0.005) | (0.005) | (0.005) | (0.005) |
| Household size | 0.002** | 0.002*** | 0.002** | 0.002* | 0.004*** |
|  | (0.001) | (0.001) | (0.001) | (0.001) | (0.001) |
| Household income | 0.000 | 0.000* | 0.000 | 0.000 | 0.000 |
|  | (0.000) | (0.000) | (0.000) | (0.000) | (0.000) |
| Land size | -0.000 | -0.000 | -0.000 | -0.000 | -0.000 |
|  | (0.000) | (0.000) | (0.000) | (0.000) | (0.000) |
| Job status | -0.014*** | -0.013*** | -0.010*** | -0.014*** | -0.012*** |
|  | (0.003) | (0.003) | (0.003) | (0.003) | (0.003) |
| Distance | 0.002 | 0.002 | 0.002 | 0.002 | 0.001 |
|  | (0.002) | (0.002) | (0.002) | (0.002) | (0.002) |
| Intercept | 0.004 | 0.003 | -0.003 | 0.004 | -0.001 |
|  | (0.009) | (0.009) | (0.009) | (0.009) | (0.009) |
| Cities fixed effects | YES | YES | YES | YES | YES |
| R-squared | 0.061 | 0.061 | 0.061 | 0.061 | 0.062 |
| Observations | 73,373 | 73,373 | 73,373 | 73,373 | 73,373 |

*Notes*: Robust standard errors are in parenthesis which are clustered at the cities level. The values in parentheses are the values of the robust standard errors for each variable. Significance code: ***p<0.01 **p<0.05 *p<0.1.

| Variables |  | Farmland farming | |  |  |
| --- | --- | --- | --- | --- | --- |
|  | (1) | (2) | (3) | (4) | (5) |
| Off-farm employment | 0.056*** |  |  |  |  |
|  | (0.004) |  |  |  |  |
| Business employment |  | -0.027*** |  |  |  |
|  |  | (0.005) |  |  |  |
| Trailing migration |  |  | -0.088*** |  |  |
|  |  |  | (0.007) |  |  |
| Time of migration |  |  |  | -0.018*** |  |
|  |  |  |  | (0.002) |  |
| Proportion of co-migrants |  |  |  |  | -0.152*** |
|  |  |  |  |  | (0.004) |
| Head gender | 0.000 | 0.006 | -0.003 | 0.006 | 0.005 |
|  | (0.004) | (0.004) | (0.004) | (0.004) | (0.004) |
| Head age | -0.008*** | -0.008*** | -0.008*** | -0.008*** | -0.008*** |
|  | (0.000) | (0.000) | (0.000) | (0.000) | (0.000) |
| Head education | 0.004** | 0.004** | 0.005** | 0.005** | 0.001 |
|  | (0.002) | (0.002) | (0.002) | (0.002) | (0.002) |
| Medical insurance | -0.019*** | -0.017*** | -0.014*** | -0.011*** | -0.014*** |
|  | (0.004) | (0.004) | (0.004) | (0.004) | (0.004) |
| Older | -0.049*** | -0.048*** | -0.045*** | -0.047*** | -0.055*** |
|  | (0.009) | (0.009) | (0.009) | (0.009) | (0.008) |
| Household size | -0.019*** | -0.022*** | -0.021*** | -0.020*** | -0.035*** |
|  | (0.002) | (0.002) | (0.002) | (0.002) | (0.002) |
| Household income | -0.000*** | -0.000*** | -0.000*** | -0.000*** | -0.000*** |
|  | (0.000) | (0.000) | (0.000) | (0.000) | (0.000) |
| Land size | -0.004*** | -0.004*** | -0.004*** | -0.004*** | -0.004*** |
|  | (0.001) | (0.001) | (0.001) | (0.001) | (0.001) |
| Job status | 0.027*** | 0.043*** | 0.003 | 0.039*** | 0.027*** |
|  | (0.006) | (0.006) | (0.006) | (0.005) | (0.005) |
| Distance | 0.019*** | 0.020*** | 0.022*** | 0.022*** | 0.025*** |
|  | (0.003) | (0.003) | (0.003) | (0.003) | (0.003) |
| Intercept | 0.664*** | 0.680*** | 0.755*** | 0.689*** | 0.721*** |
|  | (0.099) | (0.098) | (0.100) | (0.098) | (0.100) |
| Cities fixed effects | YES | YES | YES | YES | YES |
| R-squared | 0.085 | 0.083 | 0.084 | 0.084 | 0.096 |
| Observations | 73,373 | 73,373 | 73,373 | 73,373 | 73,373 |

*Notes*: Robust standard errors are in parenthesis which are clustered at the cities level. The values in parentheses are the values of the robust standard errors for each variable. Significance code: ***p<0.01 **p<0.05 *p<0.1.

| Variables |  | Farmland transfer | |  |  |
| --- | --- | --- | --- | --- | --- |
|  | (1) | (2) | (3) | (4) | (5) |
| Off-farm employment | -0.057*** |  |  |  |  |
|  | (0.004) |  |  |  |  |
| Business employment |  | 0.034*** |  |  |  |
|  |  | (0.004) |  |  |  |
| Trailing migration |  |  | 0.079*** |  |  |
|  |  |  | (0.006) |  |  |
| Time of migration |  |  |  | 0.016*** |  |
|  |  |  |  | (0.001) |  |
| Proportion of co-migrants |  |  |  |  | 0.130*** |
|  |  |  |  |  | (0.004) |
| Head gender | -0.000 | -0.006 | 0.002 | -0.006 | -0.005 |
|  | (0.004) | (0.004) | (0.004) | (0.004) | (0.004) |
| Head age | 0.008*** | 0.008*** | 0.008*** | 0.008*** | 0.008*** |
|  | (0.000) | (0.000) | (0.000) | (0.000) | (0.000) |
| Head education | 0.006*** | 0.006*** | 0.005** | 0.005** | 0.008*** |
|  | (0.002) | (0.002) | (0.002) | (0.002) | (0.002) |
| Medical insurance | 0.007* | 0.005 | 0.003 | -0.000 | 0.003 |
|  | (0.004) | (0.004) | (0.004) | (0.004) | (0.004) |
| Older | 0.036*** | 0.035*** | 0.031*** | 0.033*** | 0.040*** |
|  | (0.009) | (0.009) | (0.009) | (0.009) | (0.009) |
| Household size | 0.017*** | 0.019*** | 0.019*** | 0.018*** | 0.031*** |
|  | (0.002) | (0.002) | (0.002) | (0.002) | (0.002) |
| Household income | 0.000*** | 0.000*** | 0.000*** | 0.000*** | 0.000*** |
|  | (0.000) | (0.000) | (0.000) | (0.000) | (0.000) |
| Land size | 0.005*** | 0.004*** | 0.004*** | 0.004*** | 0.004*** |
|  | (0.001) | (0.001) | (0.001) | (0.001) | (0.001) |
| Job status | -0.013** | -0.030*** | 0.007 | -0.025*** | -0.014*** |
|  | (0.005) | (0.005) | (0.006) | (0.005) | (0.005) |
| Distance | -0.021*** | -0.022*** | -0.024*** | -0.024*** | -0.026*** |
|  | (0.003) | (0.003) | (0.003) | (0.003) | (0.003) |
| Intercept | 0.332*** | 0.316*** | 0.248** | 0.307*** | 0.280*** |
|  | (0.099) | (0.099) | (0.100) | (0.099) | (0.100) |
| Cities fixed effects | YES | YES | YES | YES | YES |
| R-squared | 0.075 | 0.073 | 0.074 | 0.074 | 0.083 |
| Observations | 73,373 | 73,373 | 73,373 | 73,373 | 73,373 |

*Notes*: Robust standard errors are in parenthesis which are clustered at the cities level. The values in parentheses are the values of the robust standard errors for each variable. Significance code: ***p<0.01 **p<0.05 *p<0.1.

## A2 Heterogeneity analysis for farmland farming and transfer

**The heterogeneous analysis results--farmland farming**

| Variables | Higher education | Lower education | Age (>=35) | Age (<35) | Larger farmland |
| --- | --- | --- | --- | --- | --- |
|  | (1) | (2) | (3) | (4) | (5) |
| Off-farm employment | 0.094*** | 0.039*** | 0.066*** | 0.030*** | 0.055*** |
|  | (0.008) | (0.005) | (0.006) | (0.005) | (0.007) |
| Business employment | -0.048*** | -0.022*** | -0.034*** | -0.036*** | -0.029*** |
|  | (0.009) | (0.005) | (0.007) | (0.006) | (0.009) |
| Trailing migration | -0.129*** | -0.059*** | -0.087*** | 0.002 | -0.075*** |
|  | (0.012) | (0.008) | (0.009) | (0.010) | (0.011) |
| Time of migration | -0.017*** | -0.019*** | -0.031*** | -0.015*** | -0.020*** |
|  | (0.003) | (0.002) | (0.002) | (0.002) | (0.003) |
| Proportion of co-migrants | -0.146*** | -0.155*** | -0.161*** | -0.135*** | -0.147*** |
|  | (0.008) | (0.005) | (0.007) | (0.006) | (0.009) |
| Variables | Smaller farmland | Higher income | Lower income | Eastern region | Middle region |
|  | (6) | (7) | (8) | (9) | (10) |
| Off-farm employment | 0.057*** | 0.065*** | 0.048*** | 0.076*** | 0.055*** |
|  | (0.005) | (0.007) | (0.005) | (0.008) | (0.010) |
| Business employment | -0.028*** | -0.032*** | -0.021*** | -0.046*** | -0.037*** |
|  | (0.005) | (0.008) | (0.006) | (0.010) | (0.011) |
| Trailing migration | -0.097*** | -0.116*** | -0.075*** | -0.127*** | -0.066*** |
|  | (0.008) | (0.013) | (0.008) | (0.015) | (0.017) |
| Time of migration | -0.018*** | -0.011*** | -0.021*** | -0.018*** | -0.024*** |
|  | (0.002) | (0.003) | (0.002) | (0.003) | (0.004) |
| Proportion of co-migrants | -0.153*** | -0.120*** | -0.163*** | -0.193*** | -0.131*** |
|  | (0.005) | (0.009) | (0.005) | (0.008) | (0.012) |
| Variables | Western region | 1^st^ tier cities | 2^nd^ tier cities | 3^rd^ tier cities | 4^th^ tier cities |
|  | (11) | (12) | (13) | (14) | (15) |
| Off-farm employment | 0.040*** | 0.083*** | 0.055*** | 0.059*** | 0.048*** |
|  | (0.007) | (0.016) | (0.006) | (0.011) | (0.007) |
| Business employment | -0.011 | -0.041** | -0.029*** | -0.034*** | -0.017** |
|  | (0.008) | (0.020) | (0.007) | (0.013) | (0.007) |
| Trailing migration | -0.070*** | -0.136*** | -0.091*** | -0.090*** | -0.078*** |
|  | (0.011) | (0.026) | (0.010) | (0.020) | (0.010) |
| Time of migration | -0.019*** | -0.012** | -0.017*** | -0.020*** | -0.022*** |
|  | (0.003) | (0.005) | (0.002) | (0.004) | (0.003) |
| Proportion of co-migrants | -0.151*** | -0.160*** | -0.157*** | -0.150*** | -0.146*** |
|  | (0.008) | (0.017) | (0.007) | (0.013) | (0.007) |
| Control variables | YES | YES | YES | YES | YES |
| Cities fixed effects | YES | YES | YES | YES | YES |

*Notes*: Robust standard errors are in parenthesis which are clustered at the cities level. All regressions control for the household and cities fixed effects. The values in parentheses are the values of the robust standard errors for each variable. Significance code: ***p<0.01 **p<0.05 *p<0.1.

**The heterogeneous analysis results--farmland transfer**

| Variables | Higher education | Lower education | Age (>=35) | Age (<35) | Larger farmland |
| --- | --- | --- | --- | --- | --- |
|  | (1) | (2) | (3) | (4) | (5) |
| Off-farm employment | -0.079*** | -0.046*** | -0.064*** | -0.035*** | -0.059*** |
|  | (0.008) | (0.005) | (0.006) | (0.005) | (0.007) |
| Business employment | 0.038*** | 0.031*** | 0.036*** | 0.041*** | 0.034*** |
|  | (0.009) | (0.005) | (0.007) | (0.006) | (0.009) |
| Trailing migration | 0.112*** | 0.056*** | 0.078*** | 0.000 | 0.074*** |
|  | (0.011) | (0.008) | (0.009) | (0.010) | (0.011) |
| Time of migration | 0.013*** | 0.016*** | 0.026*** | 0.013*** | 0.017*** |
|  | (0.003) | (0.002) | (0.002) | (0.002) | (0.003) |
| Proportion of co-migrants | 0.125*** | 0.132*** | 0.133*** | 0.118*** | 0.128*** |
|  | (0.008) | (0.005) | (0.006) | (0.006) | (0.008) |
| Variables | Smaller farmland | Higher income | Lower income | Eastern region | Middle region |
|  | (6) | (7) | (8) | (9) | (10) |
| Off-farm employment | -0.058*** | -0.060*** | -0.052*** | -0.069*** | -0.047*** |
|  | (0.005) | (0.007) | (0.005) | (0.008) | (0.010) |
| Business employment | 0.034*** | 0.030*** | 0.031*** | 0.043*** | 0.033*** |
|  | (0.005) | (0.008) | (0.006) | (0.009) | (0.011) |
| Trailing migration | 0.083*** | 0.104*** | 0.067*** | 0.113*** | 0.052*** |
|  | (0.008) | (0.013) | (0.008) | (0.015) | (0.017) |
| Time of migration | 0.015*** | 0.010*** | 0.017*** | 0.016*** | 0.022*** |
|  | (0.002) | (0.002) | (0.002) | (0.003) | (0.004) |
| Proportion of co-migrants | 0.131*** | 0.108*** | 0.137*** | 0.179*** | 0.117*** |
|  | (0.005) | (0.009) | (0.005) | (0.008) | (0.012) |
| Variables | Western region | 1^st^ tier cities | 2^nd^ tier cities | 3^rd^ tier cities | 4^th^ tier cities |
|  | (11) | (12) | (13) | (14) | (15) |
| Off-farm employment | -0.052*** | -0.085*** | -0.054*** | -0.046*** | -0.057*** |
|  | (0.007) | (0.016) | (0.006) | (0.011) | (0.007) |
| Business employment | 0.029*** | 0.042** | 0.034*** | 0.026** | 0.031*** |
|  | (0.008) | (0.019) | (0.007) | (0.012) | (0.007) |
| Trailing migration | 0.066*** | 0.140*** | 0.076*** | 0.071*** | 0.074*** |
|  | (0.011) | (0.026) | (0.010) | (0.019) | (0.010) |
| Time of migration | 0.013*** | 0.013** | 0.015*** | 0.018*** | 0.016*** |
|  | (0.003) | (0.005) | (0.002) | (0.004) | (0.002) |
| Proportion of co-migrants | 0.116*** | 0.156*** | 0.140*** | 0.128*** | 0.114*** |
|  | (0.008) | (0.017) | (0.007) | (0.012) | (0.007) |
| Control variables | YES | YES | YES | YES | YES |
| Cities fixed effects | YES | YES | YES | YES | YES |

*Notes*: Robust standard errors are in parenthesis which are clustered at the cities level. All regressions control for the household and cities fixed effects. The values in parentheses are the values of the robust standard errors for each variable. Significance code: ***p<0.01 **p<0.05 *p<0.1.
